# Validating e-learning in continuing pharmacy education: user acceptance and knowledge change

**DOI:** 10.1186/1472-6920-14-33

**Published:** 2014-02-15

**Authors:** Krzysztof Nesterowicz, Tadeusz Librowski, Samuel Edelbring

**Affiliations:** 1 Department of Radioligands, Jagiellonian University, Medical College, Faculty of Pharmacy, Medyczna 9 Street, Krakow 30-688, Poland; 2 Department of Learning, Informatics, Management and Ethics, Karolinska Institutet, Tomtebodavägen 18A, Stockholm 171 77, Sweden

**Keywords:** E-learning, Continuing pharmacy education, Lifelong learning, Just-in-time learning

## Abstract

**Background:**

Continuing pharmacy education is becoming mandatory in most countries in order to keep the professional license valid. Increasing number of pharmacists are now using e-learning as part of their continuing education. Consequently, the increasing popularity of this method of education calls for standardization and validation practices. The conducted research explored validation aspects of e-learning in terms of knowledge increase and user acceptance.

**Methods:**

Two e-courses were conducted as e-based continuing pharmacy education for graduated pharmacists. Knowledge increase and user acceptance were the two outcome measured. The change of knowledge in the first e-course was measured by a pre- and post-test and results analysed by the Wilcoxon signed–rank test. The acceptance of e-learning in the second e-course was investigated by a questionnaire and the results analysed using descriptive statistics.

**Results:**

Results showed that knowledge increased significantly (*p* < 0.001) by 16 pp after participation in the first e-course. Among the participants who responded to the survey in the second course, 92% stated that e-courses were effective and 91% stated that they enjoyed the course.

**Conclusions:**

The study shows that e-learning is a viable medium of conducting continuing pharmacy education; e-learning is effective in increasing knowledge and highly accepted by pharmacists from various working environments such as community and hospital pharmacies, faculties of pharmacy or wholesales.

## Background

The pharmacy profession has been changing continuously and pharmacists are increasingly involved in patient monitoring and consultation with other healthcare professionals as partners. Therefore, in more and more countries, continuing professional training is a requirement for pharmacists, as is for many healthcare professions, to keep their license valid. Continuing Education (CE) plays an important role in maintaining and updating pharmaceutical skills and knowledge [1]. CE courses for pharmacists, provided by professional associations, pharmacy boards, universities, teaching hospitals, and pharmaceutical companies, vary widely in their scope and breadth of content. Only a few countries (e.g. France, Japan, Poland, Singapore) have mandatory accreditation of providers of CE for pharmacists [2]. The establishment of CE standards and accreditation of providers vary between countries. In Canada, the provincial regulatory authority (provincial programmes) and the Canadian Council for Continuing Education in Pharmacy (national programmes) accredit CE courses. In Finland, each of the CE providers have set their own standards. For example, the Pharmaceutical Learning Centre and the universities provide most of the CE curricula for pharmacists and they follow their own standards. In Portugal, the quality of each CE activity is subject to evaluation by the Portuguese Pharmaceutical Society. These encompass the definition of learning objectives, programme content and educators, applicability and relevance to practice, among others [1]. All 50 states in the US require completion of CE credits for license renewal. The number of credits and renewal schedule varies by state.

In Poland the continuing education of graduated pharmacists is mandatory in order to keep their professional license valid [3]. A continuing education credit is a scale to measure one’s life-long learning activity in Poland. One continuing education credit shall be equivalent to 45 minutes of live classroom or actual lecture time, respectively for on-site or e-learning course according to the regulation of the Polish Ministry of Health [4]. Pharmacists receive credits by taking part in various campus-based or e-learning courses, training and conferences. Polish pharmacists have to collect 100 educational credits every 5 years for their professional license renewal. At least 50 of these credits must be obtained from courses that are evaluated with examinations - so called 'hard’ credits, as opposed to 'soft’ credits which one can obtain by participating in the course or conference without the need to pass a final exam. The educational process remains under control of accredited Centres of Postgraduate Education, working in cooperation with Local Pharmaceutical Chambers. Each pharmacist has his own 'educational card’ where credits are recorded [5].

More than 80% of the professionally active pharmacists in Poland (over 17 thousand professionals) take part in courses carried out with the use of e-learning platforms. We observe that the large numbers of pharmacists using e-learning platforms in Poland are from remote areas, far from academic centres. E-learning makes it possible for this group to take part in CE and save on time and travel costs, which they would need for campus-based CE.

At present, there are four available national online platforms offering accredited courses: 1) e-duk@cja, platform of Regional Pharmaceutical Chamber in Krakow (http://e-dukacja.pl/), 2) platform of Medical University of Lodz (http://www.e-umed.pl/**)**, 3) platform of Polish Pharmaceutical Society (https://www.szkolenia.ptfarm.pl/) [6], 4) platform of the Pharmaceutical School of Management (http://www.e-aptekarska.pl/). The earliest one (e-duk@cja) was implemented in February 2005 [7] and the newest (e-aptekarska.pl/) in 2013.

The Warsaw Medical University administered the online platform farmacja.edu.pl, however, there have not been accredited continuing education courses on it since 2013 [8].

All online courses distributed by online platforms e-dukacja.pl from Krakow and e-umed.pl from Lodz are free of charge. Other platforms for pharmacists in Poland distribute most of their e-courses after paying the fee (Table 1). All online courses must be provided by experts in their field in order to be accredited.

**Table 1:** E-learning platforms for pharmacists in Poland

**e-learning platform**	**Established (year)**	**Access to e-courses**	**Administrator**	**Registered pharmacists**
e-dukacja.pl	2005	Free of charge	Local Pharmaceutical Chamber in Krakow	About 19,000
e-umed.pl	2009	Free of charge	Medical University of Lodz	7,523 (18 Sept. 2013)
szkolenia.ptfam.pl	2010	Mostly charged	Polish Pharmaceutical Society	About 1,000
e-aptekarska.pl	2013	Mostly charged	Pharmaceutical School of Management	n/a

Before having access to courses from online platforms, users need to register and provide their personal data, including their local pharmaceutical chamber and pharmacy work permit number.

The e-duk@cja platform consists of the PHP web scripting language and the MySQL database server. The PHP script language can be deployed on most web servers and also used as a standalone shell on almost every platform, free of charge [9]. The reasons for choosing the PHP language were: its wide usage, simplicity and ease in rebuilding the whole system [3].

E-learning is fast becoming a part of undergraduate courses, as an adjunct to traditional learning activities [10] for pharmacy students and for pharmacology content in other healthcare professionals’ training. This blended approach might be more attractive to adult learners because of their assumed higher levels of motivation and capability for self-directed learning [11]. Specific pharmacy post-graduate level courses [3] and interprofessional online collaborations in learning and practice for healthcare professionals are also evident [12,13]. E-learning systems have become important tools in the process of the continuing education of pharmacists, especially in Europe, USA, Australia and Canada.

However, the route to using e-learning as CE is not straightforward. High setup costs and time commitments to maintain quality are issues brought up in this respect [14]. The lack of quality assurance standardization has also been pointed out [15,16]. In order to establish a foundation for quality assurance standardization, e-learning must fulfil requirements of conveying knowledge and meet acceptance with its users.

### Aims of the study

The motivation to conduct this study was to assess if e-courses were a viable method of providing continuing education in pharmacy. More specifically, the goal was to investigate pharmacists’ knowledge development and user acceptance during e-learning implementation in continuing education. Two research questions were posed to guide the study: does e-learning contribute to increasing knowledge of pharmacists and do today’s pharmacists accept e-learning as a medium for continuing education?

This study uses empirical data to report on e-learning acceptance and knowledge creation with the purpose of validating the use of e-learning in continuing pharmacy education.

## Methods

Two e-courses were used for research in the e-based continuing pharmacy education (CPE) for graduated pharmacists. Both e-courses were validated regarding their content by a scientific committee of three professors of pharmacy [5]. The first course covered aspects of modern antibiotic and chemotherapy of infectious diseases and the study conducted on it lasted five and a half months. The other covered effectiveness and safety of new classes of antidiabetic drugs and the study conducted on it lasted one month. Users who completed the first course, *Modern Antibiotic and Chemotherapy of Infectious Diseases* and passed the final knowledge test obtained 10 educational 'hard’ credits. Users of the second course, *Diabetes as a Problem of Modern Medicine – Effectiveness and Safety of New Classes of Anti-Diabetic Drugs* obtained 8 educational 'hard’ credits after passing the final test. To pass the final test of both courses correct answers on at least 60% of the questions were required.

The course about modern antibiotic and chemotherapy was divided into 5 thematic modules: 1) *Modern Antibiotic Therapy,* 2) *Herpesviruses,* 3) *Mycoses,* 4) *Dysbacterioses,* 5) *Antibiotic Therapy.* And the course about diabetes included 7 thematic modules: 1) *The Role of a Pharmacist in Prevention and Treatment of Diabetes,* 2) *Regulation of Carbohydrate Metabolism,* 3) *Complications of Diabetes,* 4) *New Perspectives of Pharmacotherapy of Type 2 Diabetes – Incretin Mimetics and Amylin Analogues,* 5) *Identification and Reducing the Risk of Hypoglycaemia,* 6) *Diabetes in Pregnancy,* 7) *Diabetes - Epidemiology, Aetiology, Symptoms, Treatment and Risk of Complications.*

The content of courses was presented online in the form of slides stored in the Adobe Flash file format. The presentation included text, numerous pictures and graphs. Attendees from both courses were simultaneously invited to the research on a voluntary basis. Access to the e-courses and research was opened for pharmacists registered on the e-learning platform e-duk@cja (about 13,000 users at that time) who were obliged to gather educational credits according to CPE regulations. There were 553 participants in the study who were from all regions of Poland, representing those who worked in community and hospital pharmacies or pharmaceutical wholesales.

Participants could communicate amongst one another and with a tutor asynchronously via online forum which is an interactive element for each course implemented on the e-learning platform. The forum is a place where any user can express feedback about the course, start a discussion or raise questions which can be seen and answered by other users or a tutor. The forum is also a place to express general comments related to the platform, to share ideas or proposals for other e-courses for pharmacists to implement there. Furthermore, every user owns an internal mailbox where he can receive messages from a tutor or send his own messages.

The studies were carried out in 2012 and 2013, respectively, and approved by Bioethical Commission of the Jagiellonian University, opinion no. KBET/235/B/2010.

### Demographics

General demographic characteristics of both cohorts are shown in Table 2.

**Table 2:** Demographics of both cohorts

**e-Course**	***Modern Antibiotic - and Chemotherapy of Infectious Diseases***	***Diabetes as a Problem of Modern Medicine - Effectiveness and Safety of New Classes of Antidiabetic Drugs***
***n*** **(completing the research)**	315	238
**Gender distribution, male : female**	68(21.6%) : 247(78.4%)	57(24%) : 181(76%)
**Average age in years (age range)**	40.8 (26–79)	40.5 (26–79)
**Standard deviation in years**	12.3	11.5

### Knowledge test

**Figure 1:**
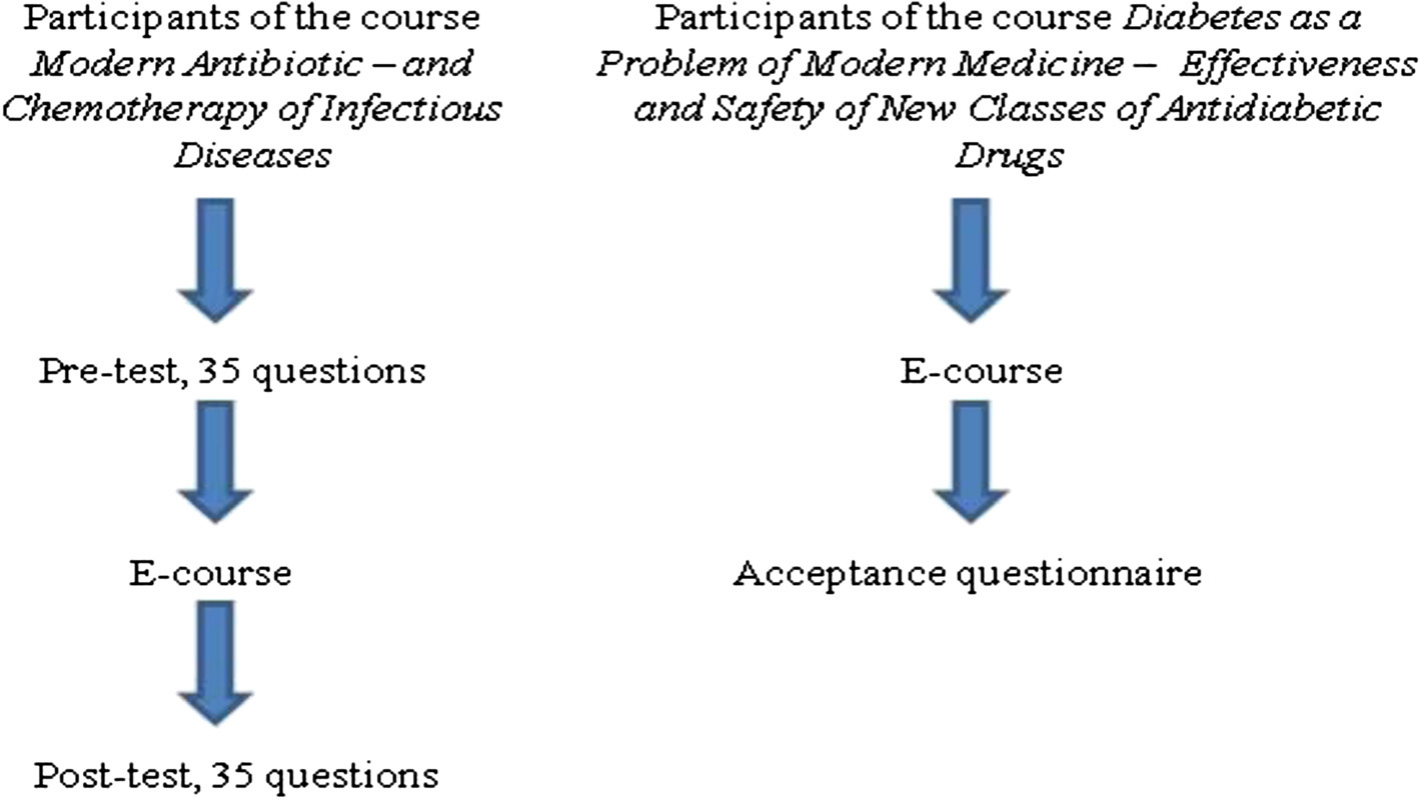
Visual representation of the study.

To assess the knowledge increase of participants of the course *Modern Antibiotic and Chemotherapy of Infectious Diseases,* an online test of 35 questions was prepared by a team of experts and used as both the pre- and post-test. Each expert independently created one module of the e-learning course and multiple choice questions related to the content of the module. All authors made sure not to inadvertently emphasise the answers to the test in the content of the e-learning course. Each multiple choice question consisted of one correct answer and three distractors. A correct answer scored one point toward the total mark of 35 points, and an incorrect answer scored zero. After completing the pre-test participants did not have access to the correct answers. The designed questions were related to the content provided by the e-course *Modern Antibiotic and Chemotherapy of Infectious Diseases* (Figure 1).

We did not intend to discourage in any way our e-course participants from using additional sources of knowledge related to the subject provided by our e-courses. We made sure that questions put in the pre- and post-tests were directly related to the content of the course but our attendees could in parallel freely obtain and deepen their knowledge via other media.

### Questionnaire on acceptance

A questionnaire assessing users acceptance of e-learning was prepared with four Likert items graded from 1-5 and free text response questions. It covered various aspects of acceptance of e-learning in Continuing Pharmacy Education (CPE) and its previous use. Free text response questions allowed attendees to add any additional feedback. The questionnaire on acceptance was provided only to participants of the e-course *Diabetes as a Problem of Modern Medicine* after completing it (Figure 1).

### Analysis

The results of the pre- and post-test knowledge change were analysed by the Wilcoxon signed–rank test. This method was selected due to the non-normality in data distribution. A critical value of *p* < 0.05 was selected in comparison between the pre- and post-test. The acceptance questionnaire was analysed using descriptive statistics. Free text responses were analysed with content analysis and summarised into categories. Statistical measurements were conducted with the use of the programme SPSS.

## Results

There were 939 attendees who signed up for the e-course *Modern Antibiotic and Chemotherapy of Infectious Diseases*. Among them 315 completed both pre- and post-test (participation rate: 34%). There were 497 pharmacists who signed up and finished the e-course *Diabetes as a Problem of Modern Medicine – Effectiveness and Safety of New Classes of Antidiabetic Drugs.* Among them 238 (participation rate: 48%) responded to the acceptance questionnaire.

### Knowledge test

**Figure 2:**
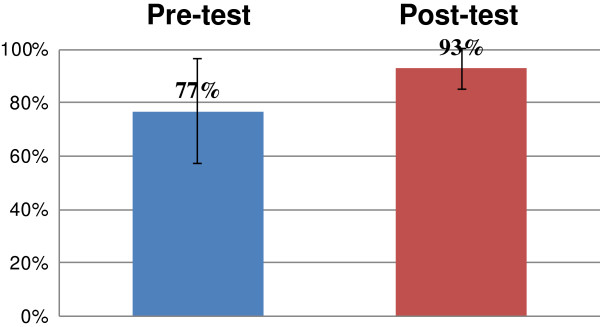
**The average results and standard deviation of the test of knowledge conducted before and after the course (*n*** **= 315,*p*** **< 0.001).**

The pharmaceutical knowledge increased significantly (*p* < 0.001) after participation in the e-course *Modern Antibiotic and Chemotherapy of Infectious Diseases*. The knowledge increased significantly by 16 pp. The average results of the pre- and post-test are presented as percentages in Figure 2.

### Questionnaire on acceptance

**Figure 3:**
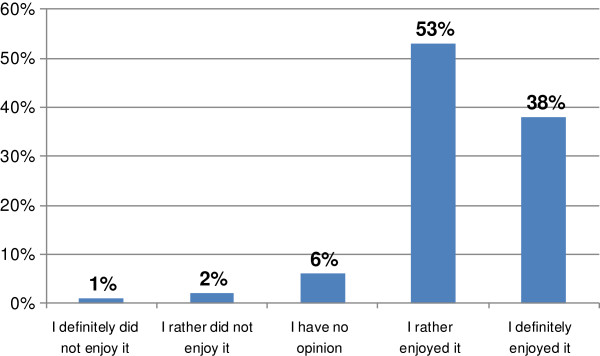
**Responses to the question: 'Did you enjoy the course?’ (*n*** **= 238).**

There were 94% of respondents (222 of 237) who had previously taken part in e-courses related to Continuing Pharmacy Education (CPE) and 97% (224 of 232) who had previously taken part in campus-based courses related to CPE. Regarding user acceptance, 91% of survey respondents (217 of 238) enjoyed the course (Figure 3).

**Figure 4:**
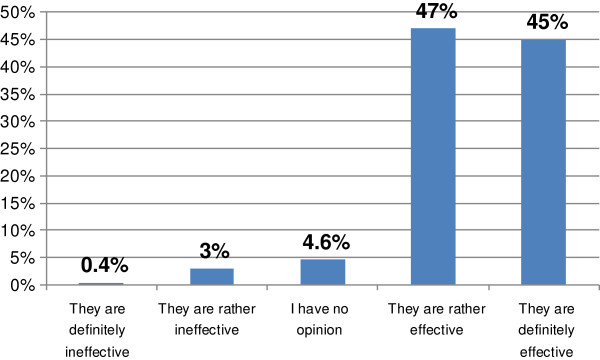
**Responses to the question: 'Please, indicate what you think about the effectiveness of such courses’ (*n*** **= 236).**

There were 92% respondents (217 of 236) of the survey who thought that e-courses were effective (Figure 4).

**Figure 5:**
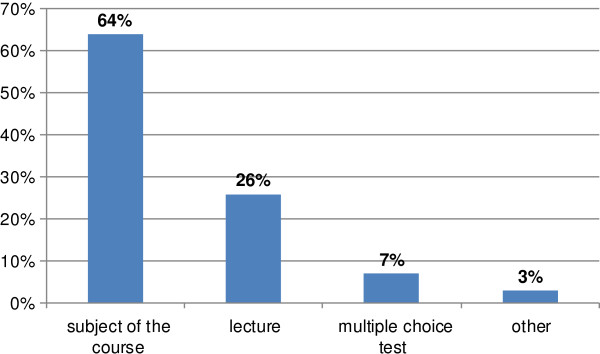
**Responses to the question: 'What did you like the most in the completed course?’ (*n*** **= 235).**

The most valued aspect was the subject of the course (Figure 5).

Open questions that required written answers (6) revealed areas of the course that needed improvement, but also showed that participants believed that it provided a convenient method of keeping up to date with current pharmaceutical practices.

**Figure 6:**
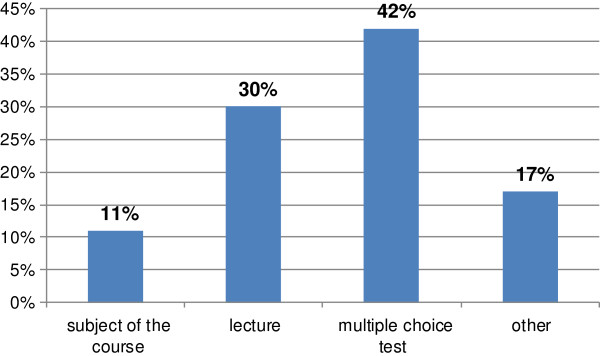
**Responses to the question: 'What did you like the least in the completed course?’ (*n*** **= 201).**

The least valued aspect in the course, according to attendees, was the form of assessing the knowledge (multiple choice test - Figure 6).

In the free text responses, 5 attendees emphasised the issue of too much information provided with the course. Similarly, 5 other pharmacists also stated the problem of the content being too detailed. However, there were 5 participants who expressed their satisfaction with the course.

## Discussion

The study sets out to assess whether e-courses can be used to increase knowledge in Continuing Pharmacy Education (CPE) and if the e-course format is accepted by pharmacists. The population represented pharmacists from community and hospital pharmacies and those employed in pharmaceutical wholesales.

The main finding of this study is the effectiveness in terms of increased knowledge (16%) by using an e-learning course. Furthermore, high levels of acceptance and satisfaction of e-courses were found among attendees. The acceptance is explicitly expressed in the questionnaire data, and also by the large number of voluntary participants. Attendees indicated the importance of this form of learning in their continuing education process. It is thus, safe to say that e-learning contributes to an increase in the knowledge of pharmacists. Furthermore, today’s pharmacists do accept e-learning as a medium for continuing education.

We found it interesting and important that the majority of the cohort (92%) stated that e-courses were effective (Figure 4). Ninety one percent of participants enjoyed the course *Diabetes as a Problem of Modern Medicine* (Figure 3). The pharmacists who responded referred to the need of such form of courses in CPE and emphasised the importance of them.

As noted by Carswell and Venkatesh [17], much of the research on e-learning has examined outcome differences between online and traditional classes [18,19] or offered experiences of teachers or students. Knowledge increase and acceptance are key issues if e-courses are to be successful tools in CPE. This study is one of the first to assess those aspects in Continuing Pharmacy Education in Poland. This research is relevant as it assesses the effectiveness of e-learning in increasing knowledge and acceptance of this way of learning. In the available literature there are rare evidences of such studies on CPE. There is one experiment conducted in 2009 and published in 2012, which explores the conveyance of skills with the use of e-learning in CPE [6]. The study compared the effectiveness of an e-learning course against a campus-based one by checking the techniques for measurement of blood pressure using a mechanical sphygmomanometer with an aneroid manometer and a stethoscope. There were, however, no significant differences in precision of blood pressure measurement between the intervention (e-learning course) or control group (campus-based course). Pharmacists trained by e-learning and on-site course showed the same level of preparation in measuring blood pressure of their patients. Consequently, e-learning can be used to convey some skills to the same effect as traditional methods.

In the atmosphere of constant changes, updates and new findings in pharmaceutical sciences, nobody doubts the need to keep the knowledge up-to-date in order to stay professionally active and reliable. More and more countries implement CPE as obligatory lifelong learning programmes. The role of e-learning in CPE has become crucial in recent years. It is a convenient way of learning, which can take place anytime and anywhere. This flexibility is particularly attractive to professionally active pharmacists who often have no time to travel and attend conventional courses in big academic centres. E-learning helps to save additional costs for participants (travel, accommodation) and the same as for providers (renting the venue, printing materials). On the other hand, there are advantages of conventional learning over e-learning, like direct interpersonal relations, live contact with the tutor, exact definite time and place of training, more transparent verification of knowledge and training of interpersonal ability. Therefore, e-courses need to be validated and standardised the same as conventional courses, to secure the level of provided information. It is not only the content of e-courses that requires reviewing by specialists, but also the way they are designed and provided to attendees.

CE e-learning courses should be validated regarding its facilitation of knowledge creation. We argue that for this scope the pre- and post-test method is relevant. Besides, we encourage implementing questionnaires on acceptance into e-courses to receive feedback from users and check the level of satisfaction. Furthermore, users should be provided with the possibility to communicate with a tutor and other users (asynchronously, like mailing list or fora and synchronously, like Internet instant messengers, e.g. Skype or Yahoo! Messenger).

The phenomenon of e-learning is very dynamic and it is difficult to predict its exact place and shape in the lifelong learning process in the near future. Undoubtedly, e-learning is going to be more relevant in the future than it is now, and play a larger role in the continuing education of healthcare professionals.

### Limitations and recommendations for future studies

The study was not designed as an experiment with a control group, but using a pre- and post-test in a real-life setting. This means that we cannot rule out the possibility that knowledge increase may result from other sources. However, the naturalistic setting contributes to increased ecological validity.

The participation rate in completing pre-and post-test was low (34%) due to the voluntary nature of the pre-test. The same situation applied to the voluntary acceptance questionnaire. The number of attendees who took part in both experiments was, however, reasonably high: 315 in the pre- and post-test and 238 in the acceptance questionnaire. Nevertheless, because of lack of available data about the differences between the respondents and the non-respondents, we cannot rule out the fact that the participants could be biased in their representation. It is reasonable to assume that pharmacists, who do not favour e-learning, will not apply voluntarily to e-courses. Future studies should thus aim to analyse reasons for non-participation in e-courses.

The current study contributes with knowledge on acceptance and increased learning which are crucial for implementing new approaches to education. Following Kirkpatrick’s model of educational evaluation, next steps involve change in behaviour and impact on practice [20]. Future studies should aim for reaching these dimensions in order to establish the effectiveness of e-learning in CPE.

Furthermore, researches are invited to compare different types of platforms and course designs. This is a first step towards increasing knowledge and acceptance for e-learning in this user group (pharmacists in CE).

E-learning enhances significantly educational opportunities for pharmacists. However, this potential requires a certain level of institutional readiness in human and infrastructural resources. One of the major tasks for pharmacy faculties and other health education providers is to find optimal methods to incorporate e-learning into the educational process [21].

## Conclusion

The study shows that e-learning is a viable medium of conducting continuing pharmacy education. E-courses are effective in increasing knowledge and highly accepted by pharmacists from various working environments, such as community and hospital pharmacies, faculties of pharmacy or wholesales. In light of the increasing use of e-learning in continuing pharmacy education, further studies are warranted, exploring not only its effectiveness in conveying knowledge but also its influence on pharmacy practice.

## Abbreviations

E-learning: Electronic learning; e-course: electronic course; CE: Continuing education; CPE: Continuing pharmacy education.

## Competing interests

The authors declare that they have no competing interests.

## Authors’ contributions

KN designed and conducted the research, collected and analysed data, drafted the manuscript. TL was involved in revising the manuscript critically and in supervising. SE was involved in drafting the manuscript, revising it critically for important intellectual content, providing consultancy in describing and interpretation of obtained results. All authors read and approved the final manuscript.

## Authors’ information

KN – MPharm, PhD candidate. Department of Radioligands, Jagiellonian University, Medical College, Faculty of Pharmacy, Medyczna 9 Street, 30–688 Krakow, Poland. TL – PhD, Associate Professor. Department of Radioligands, Jagiellonian University, Medical College, Faculty of Pharmacy, Medyczna 9 Street, 30–688 Krakow, Poland. SE – PhD, Lecturer. Department of Learning, Informatics, Management and Ethics, Karolinska Institutet, Tomtebodavägen 18A, 171 77, Stockholm, Sweden.

## Pre-publication history

The pre-publication history for this paper can be accessed here:

http://www.biomedcentral.com/1472-6920/14/33/prepub
